# Brain and testis: more alike than previously thought?

**DOI:** 10.1098/rsob.200322

**Published:** 2021-06-02

**Authors:** Bárbara Matos, Stephen J. Publicover, Luis Filipe C. Castro, Pedro J. Esteves, Margarida Fardilha

**Affiliations:** ^1^ Laboratory of Signal Transduction, Department of Medical Sciences, Institute of Biomedicine—iBiMED, University of Aveiro, 3810-193 Aveiro, Portugal; ^2^ School of Biosciences, The University of Birmingham, Edgbaston, Birmingham B15 2TT, UK; ^3^ CIIMAR/CIMAR—Interdisciplinary Centre of Marine and Environmental Research, University of Porto, Porto, Portugal; ^4^ Department of Biology, FCUP—Faculty of Sciences, University of Porto, Porto, Portugal; ^5^ CIBIO-InBIO, Research Centre in Biodiversity and Genetic Resources, Campus Agrico de Vairão, University of Porto, 4485-661 Vairão, Portugal

**Keywords:** brain, neuron, testis, sperm, molecular

## Abstract

Several strands of evidence indicate the presence of marked similarities between human brain and testis. Understanding these similarities and their implications has become a topic of interest among the scientific community. Indeed, an association of intelligence with some semen quality parameters has been reported and a relation between dysfunctions of the human brain and testis has also been evident. Numerous common molecular features are evident when these tissues are compared, which is reflected in the huge number of common proteins. At the functional level, human neurons and sperm share a number of characteristics, including the importance of the exocytotic process and the presence of similar receptors and signalling pathways. The common proteins are mainly involved in exocytosis, tissue development and neuron/brain-associated biological processes. With this analysis, we conclude that human brain and testis share several biochemical characteristics which, in addition to their involvement in the speciation process, could, at least in part, be responsible for the expression of a huge number of common proteins. Nonetheless, this is an underexplored topic, and the connection between these tissues needs to be clarified, which could help to understand the dysfunctions affecting brain and testis, as well as to develop improved therapeutic strategies.

## Graphical abstract

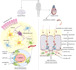

## 1. Introduction

The human body is an orchestrated set of different organs that work together, contributing to the maintenance of overall health and homeostasis. The human brain is the control center of the nervous system, playing a critical coordination role. It receives signals from sensory organs and translates them into functional information to multiple physiological compartments such as muscles and glands. In addition, the brain is also responsible for speech production, memory storage, and the elaboration of thought and emotion [1,2]. The human testis is the male gonad, and is of the utmost importance for reproduction and species evolution. It has two main functions: production of gametes (sperm) and synthesis/secretion of hormones (primarily, testosterone) [3,4].

Despite these clearly dissimilar functions and the apparent structural and morphological differences between human brain and testis, in the last four decades it has become increasingly evident that these tissues share several features. The similarity was further confirmed by analysis of gene expression, with evidence that human brain and testis, among all the organs of the body, share the highest number of genes [5,6]. More recently, authors found a positive correlation between general intelligence and three key measures of semen quality: sperm concentration, sperm count and sperm motility [7]. A possible association between male sexual dysfunction and neurological disorders was also proposed by several authors [8,9]. These findings raise some interesting questions. (i) Why do the human brain and testis share a similar gene expression profile? (ii) Have these tissues a similar cellular organization and cooperation between cell types? (iii) Are their functions related? (iv) What are the implications of the similarities between human brain and testis?

In this context, we review the similarities between human brain and testis, and between human neuron and sperm at the cellular and molecular levels. The proteomic profile of the two human tissues (brain and testis) and the two types of cells (neuron and sperm) were also compared and critically discussed.

## 2. Brain and testis

### 2.1. Cellular and molecular similarities

When human brain and testis, two apparently distinct tissues with very different functions, were compared, several similarities, spanning from molecular to cellular levels of organization, became evident. The main cellular and molecular similarities between these two organs are summarized in table 1.

**Table 1.** Cellular and molecular similarities between human brain and testis.

brain	testis
biochemical/physical support cells: astrocytes	biochemical/physical support cells: Sertoli cells
high energy demands	
metabolic cooperation: astrocytes produce lactate, which is used by neurons	metabolic cooperation: Sertoli cells produce lactate, which is used by germ cells
dependence on selenium metabolism	
high concentrations of polyunsaturated fatty acids	
highly susceptibility to oxidative damage	
blood–brain barrier	blood–testis barrier
neuroendocrine proprieties	
cytoskeleton motors (kinesins and dyneins): essential role in neuronal function	cytoskeleton motors (kinesins and dyneins): essential role in spermatogenesis

**Figure 1.**
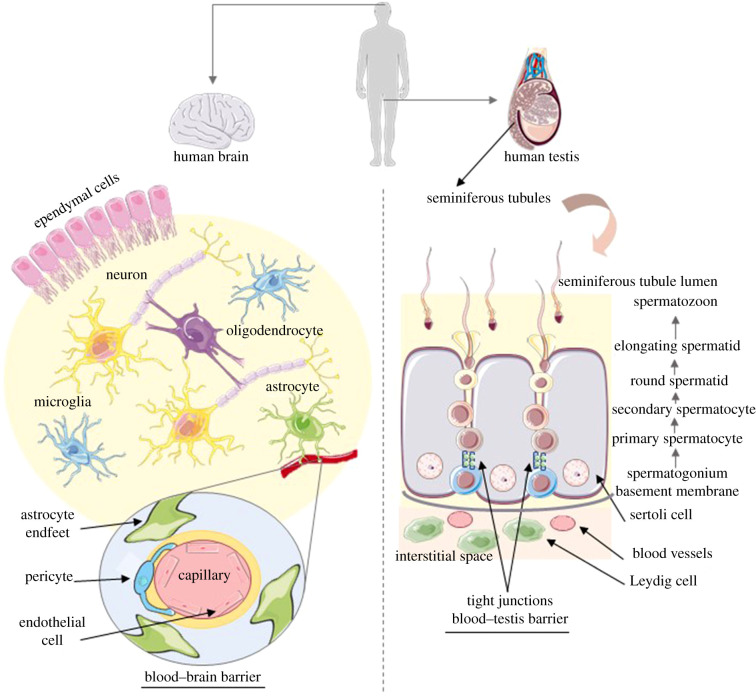
Summary of the cellular organization of human brain and testis.

Human brain and testis are both constituted by different cell types that work together to maintain the integrity and function of the tissue. Human brain is a complex and organized tissue formed mainly by neurons and support cells named glia. Neurons are the most important cells in the brain, responsible for the transmission of information. To maintain their function, glia cells are in close relation with neurons. There are four different types of glia in human brain: astrocytes, oligodendrocytes, microglia and ependymal cells, each of them essential to maintain brain function [10,11]. Likewise, testis is a well-organized tissue, composed of seminiferous tubules, in which developing germ cells and Sertoli cells are in close interaction [12]. Adjacent to the seminiferous tubules and close to the blood vessels are the Leydig cells, which produce and secrete testosterone into blood vessels [13]. The cellular organization of these two tissues is summarized in figure 1.

Astrocytes and Sertoli cells are known as the biochemical support cells of brain and testis, respectively. Beyond their important role in the metabolism of these tissues, described below, they are responsible for the physical and nutritional support of neurons and germ cells, and essential for their development and survival [14,15].

Human brain and testis are high-energy-demand tissues, executing energy-demanding processes such as cognitive functions and spermatogenesis, respectively [16]. To support these energy requirements, a metabolic cooperation between the different cell types is clear in both tissues [17,18]. In the brain, astrocytes produce lactate as a glycogen-derived product, which is transported to the neurons that use it as a preferred energy source to maintain their synaptic activity. Thus, neuronal metabolic processes are highly dependent on the activity of astrocytes [17]. Similarly, a metabolic active cooperation between developing germ cells and Sertoli cells is evident. Sertoli cells convert glucose to lactate, which is transported to and used as a central energy metabolite by developing germ cells to maintain their metabolic activity [18,19]. In addition to a similar metabolic cooperation, brain and testis both depend on selenium metabolism. A selenium-deficient diet has been associated with increased susceptibility to neurotoxicity and impaired spermatogenesis [20]. In selenium-deficient conditions, brain and testis compete for selenium utilization so that castration was associated with attenuation of neurodegeneration, mainly by increasing selenium-dependent antioxidant activity in brain [20].

Compared to other tissues, the human brain and testis are particularly susceptible to oxidative damage, due to their high energy and oxygen demand, and abundance of polyunsaturated fatty acids (PUFAs). Indeed, Kabuto *et al.* [21] exposed mice to bisphenol A, an oxidative stress inducer, during embryonic/fetal life and infancy, and collected several tissues, finding a particular underdevelopment of brain and testis, caused by increased oxidative injury. Furthermore, brain and testis have the lowest transcriptional levels of oxidative stress-related genes (for example, the gene that encodes catalase), compared to other tissues [16]. To counteract their high susceptibility to oxidative stress, these two tissues have specific blood–tissue barriers, called the blood–brain and the blood–testis barrier [22,23]. An essential role of high concentrations of PUFAs in human brain and testis function and/or development has been reported [24,25]. In the brain, the most abundant PUFA is docosahexaenoic acid (DHA), which it is mainly located at the synaptic terminals of neurons, playing a central role in neurodevelopment, function and maintenance [24]. The human germ cell line has an active lipid metabolism and displays stage-specific differences in fatty acid pattern. The inverse correlation found between the percentage of abnormal sperm and the percentage of DHA suggests a role of PUFAs in sperm morphology and development [25].

In recent decades, the Leydig cells of the human testis have been recognized as members of the neuroendocrine system. The synthesis and release of a large number of biologically active substances that are typical for nerve and neuroendocrine cells has revealed that Leydig cells are neuroendocrine cells [26]. Indeed, several neuron-specific peptides and proteins, such as Substance-P [27], synaptophysin and neural cell-adhesion molecule, have been detected in human Leydig cells [28]. Some glial-cell-specific antigens—(glial fibrillary acidic protein (GFAP), galactocerebroside (GalC), cyclic 2′,3′-nucleotide-3′-phosphodiesterase (CNPase), A2B5-antigen and O_4_-antigen, which are considered to be marker molecules of astrocytes and oligodendrocytes—were also found in Leydig cells of human testis [26]. Besides Leydig cells, Sertoli cells also express some neuron- and glial cell-specific proteins. In fact, the three isoforms of neurofilament proteins, and GFAP, GalC and CNPase were found in both Leydig and Sertoli cells of human testis [26,29].

Cytoskeleton motors, including myosin, kinesins and dyneins, play essential roles in the brain, namely in neuron polarization, extension, shape and neurotransmission processes [30]. Motor proteins also play key roles in the formation of mature sperm [31]. Spermatogenesis includes several mitotic and meiotic divisions, for which the role of motor proteins in spindle organization, chromosome congression, chromatid separation, among others, are clear. Also in the final step of spermatogenesis, called spermiogenesis, kinesins seems to be crucial in acrosome biogenesis, nuclear shaping, tail formation, and spermatid maturation and transcription [31]. The vital role of these cytoskeleton motor proteins in brain and testis function is evident by several neurodegenerative and reproductive diseases that arise from mutations or other dysfunctions of these proteins in brain and testis, respectively [32,33].

### 2.2. Proteomic comparison

According to the apparent cellular and molecular similarities between human brain and testis, it has become clear that these tissues have a similar gene expression pattern. In a UniGene pilot investigation carried out by Guo *et al.* [5], the expression data of 760 human UniGenes in 17 tissues were retrieved and compared. Unexpectedly, among the 17 tissues compared, the highest similarity in gene expression patterns was between human brain and testis with a total of 364 shared expressed UniGenes [5]. According to this study, a large-scale analysis of the expression of 33 689 genes in 15 human tissues revealed that human brain and testis shared the greatest similarity in gene expression [6]. In addition, these authors demonstrated that the similarity of gene expression between brain and testis is not exclusive to humans and may be widely present in other mammals, including rodents [6]. Several authors have demonstrated that some genes are highly or selectively expressed in brain and testis of mice (*Tb-rbp*, *Gpr37*, *Hst-1/Fgf-4*) and rat (*Ugt1a6*, *Glutx1*, *α4-b*, *Lancl1*, *Nep*) [34–41]. Moreover, Danielsson *et al.* [42] found that human brain and testis share the highest number of group-enriched genes. Although transcriptomic profiling has become a standard approach to understand the (dys)function of tissues, it is also important to evaluate how gene expression relates to the proteins that are actually being expressed. To that purpose, it is possible to use proteomics, which gives information about protein composition of a cell, tissue or organism [43].

**Figure 2.**
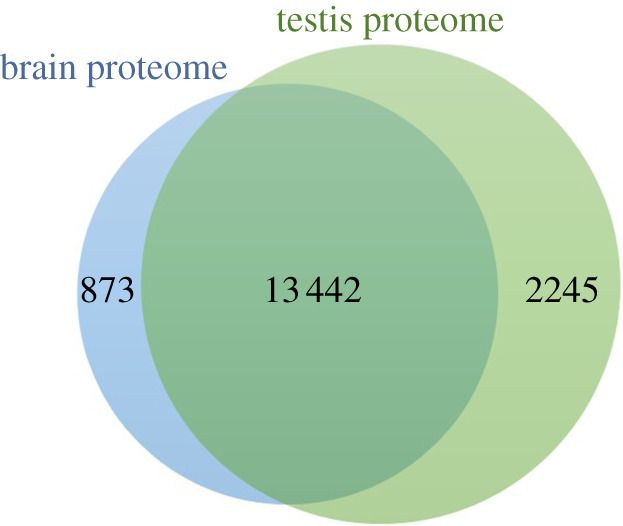
Veen diagram demonstrating the overlap between the human brain and testis proteome (based on the Jveen tool). The data of human brain and testis proteome were collected on 20 August 2019.

Herein, we compared the brain and testis proteome with that of 31 other tissues, representing all major tissues in the human body (heart, skeletal muscle, adrenal gland, parathyroid gland, thyroid gland, lung, gastrointestinal tract, salivary gland, oesophagus, stomach, duodenum, small intestine, colon, bone marrow, lymph node, spleen, appendix, pancreas, kidney, liver, gallbladder, epididymis, seminal vesicle, prostate, breast, cervix, endometrium, ovary, placenta, adipose tissue and skin), using the Human Protein Atlas (HPA) (available at www.proteinatlas.org) and the Jveen tool (available at http://jvenn.toulouse.inra.fr). The HPA is a programme that aims to map all the proteins in cells, tissues and organs using the integration of various technologies (e.g. antibody-based imaging, mass spectrometry-based proteomics, systems biology). Consistent with the gene expression analysis mentioned in the previous section [5,6], the highest number of common proteins was observed between brain and testis, suggesting that human brain and testis are the most similar tissues of the human body. The common proteins between these two tissues were retrieved and, to prevent redundancy, all proteins were annotated using the UniProtKB/Swiss-Prot accession number. From the total of 14 315 and 15 687 proteins that constitute the human brain and testis proteome, respectively, 13 442 are common to both tissues (figure 2; electronic supplementary material, table S1).

From the 13 442 common proteins between human brain and testis, we decided to highlight the proteins that are highly expressed in these two tissues, when compared with other human body tissues. To do that, we cross-checked the information from HPA with GeneCards (available at https://www.genecards.org/) and identified a total of 29 proteins highly expressed in brain and testis (table 2). To better understand the similarities between human brain and testis, we decided to explore the biological processes in which these 29 proteins are involved, using UniProt which is summarized in table 2. The analysis of protein-associated biological processes revealed specific roles of some proteins in brain and testis function and/or development. Since brain plays a key role in the control of testis function, particularly by the secretion of gonadotropin-releasing hormone (GnRH), luteinizing hormone (LH) and follicle-stimulating hormone (FSH) by the hypothalamus and pituitary, we expected more common specific proteins involved in testis function/development. Counterintuitively, 31% of the proteins are involved in brain function development, as opposed to 7% of testis function/development-related proteins.

**Table 2.** List of proteins highly expressed only in brain and testis, along with their UnitProt ID, gene name and the main biological process(es) associated (according to UnitProt). The biological processes associated with brain or testis function/development are bolded. CNS, central nervous system.

UnitProt ID	gene name	protein name	biological processes
Q9H172	ABCG4	ATP-binding cassette sub-family G member 4	cellular response to leukaemia inhibitory factor; cholesterol efflux; transmembrane transport
Q96M02	C1ORF90	(E2-independent) E3 ubiquitin-conjugating enzyme FATS	protein polyubiquitination and stabilization; regulation of centriole replication
Q13536	C1ORF61	protein CROC-4 (contingent replication of cDNA 4)	positive regulation of transcription by RNA polymerase II
Q5T035	C9ORF129	putative in characterized protein C9orf129	—
P08912	CHRM5	muscarinic acetylcholine receptor M5	chemical synaptic transport; dopamine transport; transmission of nerve impulse
Q12926	ELAV2	ELAV-like protein 2	mRNA splicing, via spliceosome; regulation of transcription
Q49AJ0	FAM135B	protein FAM135B	cellular lipid metabolic process
P43080	GUCA1A	guanylyl cyclase-activating protein 1	cellular response to calcium ion; signal transduction; visual perception
Q8NE63	HIPK4	homeodomain-interacting protein kinase 4	histone phosphorylation; peptidyl-serine phosphorylation; protein autophosphorylation
A6NGN9	IGLON5	IgLON family member 5	—
Q7Z553	MDGA2	MAM domain-containing glycosylphosphatidylinositol anchor protein 2	spinal cord motor neuron differentiation
P60323	NANOS3	Nanos homolog 3	germ cell development; multicellular organism development; regulation of cell cycle; spermatogenesis
O14594	NCAN	neurocan core protein	cell adhesion; CNS development; chondroitin sulfate biosynthetic process
Q9NQ35	NRIP3	nuclear receptor-interacting protein 3	—
Q9Y5K3	PCYT1B	choline-phosphate cytidylyltransferase B	**spermatogenesis**; phosphatidylcholine biosynthetic process
P01213	PDYN	proenkephalin-B	**chemical synaptic transmission**; G protein-coupled receptor signalling pathway; **neuropeptide signalling pathway**
Q96PV4	PNMA5	paraneoplastic antigen-like protein 5	positive regulation of apoptotic process
Q8WY54	PPM1E	protein phosphatase 1E	cellular response to drug; negative regulation of protein kinase activity
Q33E94	RFX4	transcription factor RFX4	positive regulation of transcription by RNA polymerase II; cilium assembly
Q8N6R1	SERP2	stress-associated endoplasmic reticulum protein 2	endoplasmic reticulum unfolded protein response; protein glycosylation; protein transport
Q6ZV89	SH2D5	SH2 domain-containing protein 5	—
Q99963	SH3GL3	endophilin-A3	**CNS development**; endocytosis; **positive regulation of neuron differentiation**
Q8TF17	SH3TC2	SH3 domain and tetratricopeptide repeat-containing protein 2	**peripheral nervous system myelin maintenance**; regulation of intracellular protein transport
Q8N5S1	SLC25A41	solute carrier family 25 member 41	—
Q99726	SLC30A3	zinc transporter 3 (ZnT-3)	regulation of sequestering zinc ion; response to zinc ion
O00570	SOX1	transcription factor SOX1	cell differentiation; **CNS development;** chromatin organization; **forebrain neuron development; neuron differentiation**
Q16650	TBR1	T-box brain protein 1	**brain development**; cell fate specification; **regulation of axon guidance**; regulation of transcription
O95409	ZIC2	zinc finger protein ZIC 2	**brain development**; cell differentiation; positive regulation of transcription
O96T25	ZIC5	zinc finger protein ZIC 5	cell differentiation; **CNS development**

### 2.3. Why do brain and testis appear to have similar proteomes?

The increasing evidence for similarity between the human brain and testis gene expression and protein composition raises the question of the importance of these findings. It has been hypothesized that the testis could participate in human speciation along with the brain and placenta, which may contribute to the expression of the same set of genes in both tissues [44]. It has been suggested that evolutionary changes in gene expression contribute to most of the phenotypic differences between species, but how these gene expression patterns might be passed down to the offspring is a misunderstood topic [6,45]. The involvement of testis, along with the brain and placenta, in speciation was first suggested by Wilda *et al*. [44] and the hypothalamus–pituitary–testis axis was proposed to be implicated in maintaining the similar gene expression between brain and testis [6]. Indeed, testis has been proposed as the hotspot for the appearance of new genes, which are the raw material for the evolution of species [46]. Sperm seem to be the motor of speciation. On one hand, sperm competition, that is the competitive process between sperm of different males to fertilize the same egg, is important in the formation of new species. On the other hand, sperm is also crucial to maintain the integrity of a species, by acting as a reproductive isolation barrier that precludes gene flow between species. In fact, male hybrids, characterized by the combination of two different species, seem to produce significantly fewer mature spermatozoa due to incompatibilities in the last stages of sperm development [47]. The high and specific expression of fragile X mental retardation 1 gene (*Fmr1*) in brain and testis suggests that speciation recruits the same set of tissue-specific genes that are active in those organs that are important for speciation [44].

More recently, 60 new protein-coding genes that originated de novo in the human lineage since its divergence from chimpanzee (human-specific genes) were identified [48]. These proteins became fixed in the human population, and the high levels found in testis are also in agreement with the role of this organ in the transmission of gene expression patterns to the offspring [48]. The highest expression levels in cerebral cortex and testis suggested that these genes may contribute to phenotypic features that are exclusive of humans, such as the improved cognitive ability. Indeed, human-specific *NOTCHNL2* genes were associated with a role in cortex development and neurogenesis, and have been proposed as a driving force in the evolution of human large brains [49]. Additional evidence seems to suggest that brain and testis function-associated genes are changing unusually quickly, becoming the most divergent genes between species [50].

The similarities between the human brain and testis proteome seems to be reflected in an apparent association between the (dys)function of these tissues. Indeed, an association was observed between the degenerative process in the central nervous system and testicular degeneration, without coexisting hypophyseal lesions [51]. In addition, mutations in X-linked aristaless-related homeobox gene (*Arx*) were associated with the X-linked lissencephaly with abnormal genitalia (XLAG) syndrome, a disease characterized by simultaneous microcephaly and hypogonadism [52]. Evidence in mouse suggested that alterations in the same protein may be simultaneously responsible for brain and testis dysfunction. Inactivation of Huntington disease gene (*Hdh*) in mouse brain and testis results in a progressive degenerative neuronal phenotype, along with sterility [53], while mutations in *Arx* caused abnormal development of forebrain and testis [52]. Moreover, a negative correlation was observed between testis volume and parental behaviour and nurturing-related brain activity [54].

## 3. Neuron and sperm

### 3.1. Cellular and molecular similarities

The morphology, genomic activity and function of human neuron and sperm are as different as any other two cells in the body [55]. Sperm is a very distinct cell, compared to other cells in the human body, mainly because it is a haploid cell and virtually devoid of transcription and translation [56]. However, beyond the similarities between brain and testis, several bodies of evidence of the similarities between human neuron and sperm, the fundamental units of these tissues, have been reported and are summarized in table 3.

**Table 3.** Cellular and molecular similarities between human neuron and sperm.

neuron	sperm
activate other cells: neurons or somatic effectors	activate other cells: oocyte
exocytosis of neurotransmitters in the synaptic space (essential for neuron function)	acrosomal exocytosis at the oocyte surface (essential for sperm function)
synaptic vesicles	acrosome
high concentrations of PUFAs	
presence of ‘neuronal’ receptors	
excitable cells	
presence of Ca^2+^ channels	
Ca^2+^ signalling involved in regulation of key functions	
common signalling pathways	

Both neuron and sperm can activate other cells, though the activation mechanisms involved are different. After the plasma membrane interaction of sperm with oocyte, the sperm activates the oocyte and triggers a signal transduction cascade that ultimately results in the conversion of the oocyte to a diploid embryo [57]. Neurons also have the capacity to activate other cells, namely other neurons or somatic effector cells, through chemical synapses or gap junctions (electrical synapses), not requiring contact between cells [57].

Human neuron and sperm seem also to share similarities in exocytic process. Exocytosis is a central process to their individual abilities to carry out their functions. Several components of the neuronal synaptic vesicle exocytotic machinery have been found in sperm, notably including an intricate system of plasma membrane proteins, like synaptotagmins and SNARE complex [58–61]. Neurons use exocytosis for neurite outgrowth and to release neurotransmitters from synaptic vesicles, which is essential for communication between neurons [62]. The synaptic vesicles can be compared to the acrosome of sperm, which essentially contains hydrolytic enzymes and other important fertilization factors. These enzymes are released from the sperm through a specialized form of exocytosis. This process includes membrane loss and is necessary for zona pellucida breakdown and consequent sperm–oocyte fusion [55,63]. Despite the similarities of the exocytotic process in neurons and sperm, in sperm this event only occurs once, in contrast to the continuous exocytotic activity of a neuron [55].

After the release of neurotransmitters at the synaptic gap, they interact with post-synaptic receptors (‘neuronal’ receptors) to induce or inhibit neurotransmission. Several types of ‘neuronal’ receptors, like glutamate and gamma-aminobutyric (GABA_A_), glycine and nicotinic acetylcholine receptors, have been found in sperm [64–66]. Also in sperm, the ‘neuronal’ receptors play vital roles for its normal function, including in sperm acrosomal reaction, capacitation and motility [55,67]. Due to the presence of various voltage-gated ion channels and several ligand-gated receptor channels, involved in rapid membrane potential changes, neurons are considered excitable cells [68]. In sperm, diverse types of high- and low-voltage-activated channels have been reported, suggesting that sperm may, like neurons, be considered an excitable cell [69,70].

Calcium (Ca^2+^) signalling is central to the regulation of function in both neuron and sperm cells. These distinct cell types both need to generate precisely timed and localized [Ca^2+^]_I_ signals. It appears that this has resulted in some striking similarities in the ways in which their Ca^2+^ signalling toolkits are employed [55]. Though all cells express a Ca^2+^ signalling toolkit, the types, locations and combinations of channels and pumps can vary significantly between cell types, because they are adapted to the requirements of the cell and its activities. Both neuron and sperm Ca^2+^ signalling toolkits involve a diverse range of components (including Ca^2+^-permeable channels and Ca^2+^ pumps) in both the plasma membrane and intracellular membranes, though the diversity in sperm is low in comparison to that of neurons [67,71]. In neurons, Ca^2+^ signalling is involved in the regulation of various key functions, including transmission, processing and storage of information [72]. For instance, synaptic neurotransmitter secretion, modulation of synaptic efficacy (underlying memory formation) and excitability of the neuronal membrane (the ease with which a nerve impulse can be induced) are all dependent on or modulated by [Ca^2+^]_I_ [73]. In mature sperm cells, Ca^2+^ signalling is arguably at least as important as in neurons, playing central roles in the regulation of motility and capacitation (post-ejaculatory acquisition of fertilizing ability) [71]. The complex signalling pathway that leads to acrosome reaction also requires mobilization of Ca^2+^ stores within the acrosome [74]. The best-characterized Ca^2+^ channel in sperm is CatSper, which is a sperm-specific channel essential for hyperactivated motility [75,76]. The influx of extracellular Ca^2+^ is also required for acrosome reaction, though the involvement of CatSper here is unclear [69].

Several signalling pathways are common to neuron and sperm, and seem to play essential roles in both cell types. For instance, anandamide (AEA) signalling seems to modulate human sperm motility [77]. A role as a modulator of synaptic function was also described for AEA signalling pathway [78]. In addition, Wnt signalling occurs also in both cell types where it controls both sperm maturation and neuronal differentiation [79,80]. The mTOR signalling pathway was also associated with crucial events in both neuron and sperm. Indeed, mTOR signalling regulates sperm quality in older men and is important for normal neuronal growth [81,82].

### 3.2. Proteomic comparison

**Figure 3.**
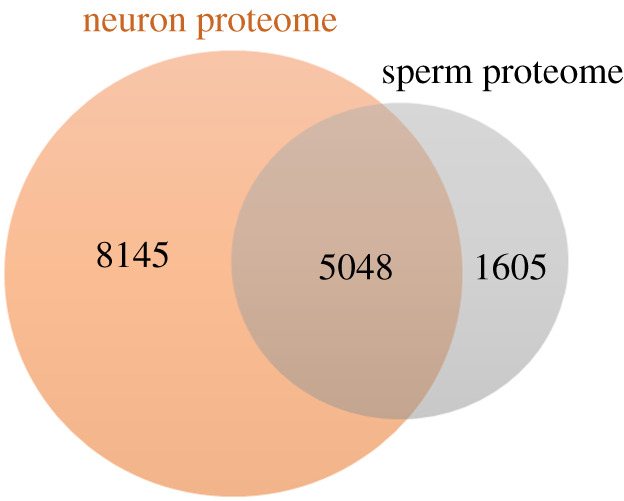
Venn diagram demonstrating the overlap between the neuron and sperm proteome (based on the Jveen tool).

The sperm proteome was recently extracted, using the PubMed database, by Santiago *et al.* [83]. To avoid redundancy, from the total list of sperm proteins, we excluded duplicates and only reviewed proteins (annotated using the UniProtKB/Swiss-Prot accession number) were considered (electronic supplementary material, table S2). Based on the same criteria, the neuronal cells proteome was retrieved from HPA. To avoid redundancy, duplicates and unreviewed proteins (according to UnitProt) were excluded. A list of all neuron proteins (available at 31 March 2021) were obtained (electronic supplementary material, table S2). A total of 13 193 and 6653 reviewed proteins constitute the neuron and sperm proteomes, respectively. A Venn diagram analysis was conducted using the Jveen tool to recover the common proteins between these two cell types. From the total proteins, 5048 are common to both human sperm and neuron (electronic supplementary material, table S2; figure 3).

From the 5048 common proteins in human neuron and sperm, a sublist was made considering the proteins with elevated expression in neuronal cells, according to HPA (www.proteinatlas.org). This analysis results in a total of 682 common proteins. Considering these common proteins, a GO analysis (using STRING: functional protein association networks) was performed and revealed a total of 328 GO terms significantly enriched, with an FDR < 0.05 (electronic supplementary material, table S2). In table 4, we summarize some of the most important biological processes in the context of the present study, together with the number of proteins associated with the GO term and the respective FDR of the annotation.

**Table 4.** Main biological processes associated with the common proteins between human sperm and neuron. A list of all the associated biological processes are found in electronic supplementary material, table S2.

GO term	description	count in gene set	FDR
development			
GO:0032 502	developmental process	268/5401	4.08 × 10^−9^
GO:0048869	cellular developmental process	175/3533	2.61 × 10^−5^
GO:2000026	regulation of multicellular organismal developmental	90/1876	2.23 × 10^−2^
GO:0021700	developmental maturation	17/216	3.35 × 10^−2^
GO:0048639	positive regulation of developmental growth	14/165	3.93 × 10^−2^
nervous system development			
GO:0007399	nervous system development	181/2206	1.11 × 10^−23^
GO:0022008	neuron projection development	68/616	6.03 × 10^−13^
GO:0048666	neuron development	76/758	1.11 × 10^−12^
GO:0061564	axon development	47/377	1.28 × 10^−10^
GO:0007417	central nervous system development	60/861	3.14 × 10^−5^
GO:0007420	brain development	47/650	1.80 × 10^−4^
GO:0021695	cerebellar cortex development	7/49	3.63 × 10^−2^
brain/neuron-associated processes			
GO:0030182	neuron differentiation	86/940	1.64 × 10^−12^
GO:0010975	regulation of neuron projection development	47/443	1.22 × 10^−8^
GO:0007411	axon guidance	27/220	4.38 × 10^−6^
GO:009893	axonal transport	9/43	1.50 × 10^−3^
GO:0008038	neuron recognition	8/34	1.80 × 10^−3^
GO:0001764	neuron migration	14/118	3.70 × 10^−3^
GO:0007158	neuron cell–cell adhesion	5/14	7.50 × 10^−3^
GO:0019228	neuronal action potential	6/31	2.17 × 10^−2^
GO:0036514	dopaminergic neuron axon guidance	3/5	2.87 × 10^−2^
exocytosis			
GO:0017156	calcium ion regulated exocytosis	12/74	9.00 × 10^−4^
GO:0016079	synaptic vesicle exocytosis	11/64	0.0011
GO:0006904	vesicle docking involved in exocytosis	6/38	0.0439
cell signalling			
GO:0007267	cell–cell signalling	95/1073	4.86 × 10^−13^
GO:0035637	multicellular organismal signalling	21/110	1.89 × 10^−7^
GO:0023052	signalling	232/5108	1.10 × 10^−4^
GO:0007215	glutamate receptor signalling pathway	9/43	1.50 × 10^−3^
GO:1905114	cell surface receptor signalling pathway	30/383	1.80 × 10^−3^
GO:1990034	calcium-mediated signalling	13/132	2.02 × 10^−2^
GO:0035556	intracellular signal transduction	76/1528	2.17 × 10^−2^
GO:0016055	Wnt signalling pathway	22/303	2.41 × 10^−2^

Among the common proteins between human neurons and sperm, several GO terms related to cell/tissue development were significantly enriched, suggesting that both cells play important roles in human tissue development. Also comparing neuron and sperm proteomes, it is observed that there are many common proteins involved in brain/neuron development and function. As expected by the important role of exocytosis in both sperm and neuron function as discussed above (table 4), both neuron and sperm express a huge number of proteins involved in exocytic process. Cell signalling-associated biological processes were also highlighted in this analysis, corroborating the idea that sperm and neuron share several important signalling pathways (table 4).

## 4. Concluding remarks

Human brain and testis share several molecular characteristics, which are reflected in a very similar proteomic profile. Our *in silico* analysis revealed that, surprisingly, human brain and testis have the highest number of common proteins, compared with other human body tissues. The common proteins are mainly involved in the function and/or development of brain, rather than in testis-associated processes. The human neuron and sperm are very distinct cells; however, they share several molecular features, and a huge number of proteins are common to both cells, mainly those involved in exocytotic and cell signalling processes, tissue development and brain/neuron-associated processes.

The similarity between human brain and testis may be explained by a biochemical convergence and by the involvement of these two tissues in the speciation process. The high similarity of proteins between human brain and testis may have clinical relevance. Indeed, the common proteins may be associated with the simultaneously impairment of brain and testis function. The identification of these proteins, along with the analysis of their role in brain and/or testis function, could help in better understanding the pathophysiology of these conditions, as well as in the development of new therapeutic strategies for treating brain or testis diseases.

## Data accessibility

This article has no additional data.
